# Associated factors for surgical site infection with spinal instrumentation surgery

**DOI:** 10.1038/s41598-025-24209-y

**Published:** 2025-11-18

**Authors:** Shinji Tanishima, Tokumitsu Mihara, Chikako Takeda, Satoshi Fujiwara, Hideki Nagashima

**Affiliations:** Division of Orthopedic Surgery, Department of Sensory and Motor Organs, School of Medicine, Faculty of Medicine, Tottori University, 36-1 Nishi-cho, Yonago, 683-8504 Tottori Japan

**Keywords:** Surgical site infection, Spinal instrumentation surgery, Emergency surgery, Associated factor, Health care, Risk factors

## Abstract

Surgical site infection (SSI) after spinal instrumentation surgery is a serious complication that is difficult to eliminate. We examined the associated factor for SSI in our institution. This retrospective study was conducted at a single institution. We included 483 patients aged ≥ 18 years who underwent spinal instrumentation surgery at our hospital between January 2016 and March 2021 and who were available for follow-up for at least 30 days. Patient factors were age, sex, malignant disease, diabetes, skin disease, hemodialysis, smoking, steroid and immunosuppressive drug use, BMI, albumin, total protein, CRP, American Society of Anesthesiologists (ASA) classification, and nasal culture, whereas surgery-related factors were re-operation, scheduled/emergency surgery, dural injury, number of fusion segments, fixation over the pelvis, vancomycin spray, operative time, and blood loss. Statistical analysis was performed using univariate analysis in the infected and uninfected groups and multivariate logistic analysis for factors showing an associated trend (*P* < 0.2). Eleven of 483 patients (2.3%) were infected, with nine having deep infections and two having superficial infections. In the univariate analysis, the patient factors were albumin level (4.1 ± 0.6: 3.7 ± 0.8 mg/dL) and CRP level (0.74 ± 1.8: 3.2 ± 5.3 mg/dL) in the uninfected group and emergency surgery rate (27.8%: 63.6%) in the infected group. Logistic analysis identified emergency surgery as a risk factor for SSI (odds ratio: 4.6, 95% confidence interval: 1.3–15.9, *P* = 0.016). Although not statistically significant, SSIs tend to recur sequentially within 3 months. Currently, SSI countermeasures are strictly implemented at all facilities, including our hospital. Thus, diabetes mellitus and immunosuppressive drugs were not risk factors, but emergency surgery was an associated factor, which may be due to the lack of sufficient infection control measures in emergencies. Once SSIs occur, they recur sequentially, suggesting that SSIs may result from complications in perioperative management.

**Supplementary Information:**

The online version contains supplementary material available at 10.1038/s41598-025-24209-y.

## Introduction

Surgical site infections (SSIs) are a significant complication in spinal instrumentation surgery, with incidence rates ranging from 2% to 10% globally^1,2^. These infections can lead to prolonged hospital stays, increased healthcare costs, and considerable morbidity and mortality^3^. The nature of spinal instrumentation surgery, involving extended operative times, substantial blood loss, and complex procedures, makes it particularly susceptible to infections^4^. Despite advances in surgical techniques and perioperative care, the complete prevention of SSIs remains challenging^5^.

Patient factors, such as diabetes mellitus, hypoalbuminemia, elevated preoperative C-reactive protein (CRP) levels, and immunosuppressive therapy are associated with increased infection risks^6,7^. Additionally, surgical factors, including prolonged operative time, multilevel fusions, and extensive blood loss, further elevate SSI risk^8,9^. To mitigate these risks, infection prevention strategies, such as perioperative antibiotic protocols and optimized surgical techniques, have been successfully implemented in elective surgeries^10^.

However, several critical gaps in our understanding persist. Emergency surgeries present unique challenges due to the limited time available for preoperative preparation and infection prevention. Although emergency procedures are a potential risk factor for SSIs, their specific role in spinal instrumentation surgery remains underexplored^11^. Furthermore, although SSI rates have seasonal variations in other surgical fields, their relevance to spinal surgeries is not well-documented^12,13^. The temporal dynamics of infection occurrences, including clustering and recurrence patterns, are also poorly understood, highlighting the need for focused studies to optimize infection control practices, particularly in high-risk situations, such as emergency surgeries.

This study aimed to investigate associated factors and temporal patterns of surgical site infections (SSIs) following spinal instrumentation surgery, to provide evidence-based insights for infection prevention.

## Subjects and methods

We included 483 patients aged ≥ 18 years (mean age of 68.9 ± 14.3 years) who underwent spinal instrumentation surgery at our hospital between January 2016 and March 2021 and who were available for follow-up for at least 30 days.

As a measure against infection, nasal culture was performed preoperatively whenever possible, and mupirocin ointment was applied to methicillin-resistant staphylococcus aureus (MRSA).

carriers. Antibiotics were administered within 30 min from the start of surgery, with additional doses administered every 3 h thereafter. Moreover, diluted povidone-iodine was used for washing intraoperatively, following the CDC guidelines^10^. Closure was performed using an antibacterial suture containing trichloroacetic acid to prevent infection^14^. During this period, perioperative antibiotic administration methods and surgical site disinfection procedures remained consistent.

Patient factors were age, sex, malignant disease, diabetes, skin disease, hemodialysis, smoking, steroid and immunosuppressive drug use, body mass index (BMI), albumin, total protein, C-reactive protein (CRP), American Society of Anesthesiologists (ASA) classification, and nasal culture, whereas surgery-related factors were re-operation, scheduled/emergency surgery, dural injury, number of fusion segments, fixation over the pelvis, vancomycin spray, operative time, and blood loss.

This retrospective study was approved by Ethical review board, Faculty of Medicine Tottori university (approval No. 21A059). All methods were performed in accordance with the relevant guidelines and regulations. Informed consent was obtained using the opt-out method in accordance with the ethical guidelines for medical research involving human subjects in Japan. For patients who were alive during the study period, consent to participate was considered obtained unless they or their families explicitly declined. For patients who had died before the study period, the Ethics Committee determined that their data could be included unless their families objected under the opt-out policy. In practice, no families declined participation. Therefore, the requirement for written informed consent was waived by the Ethics Committee.

### Statistical analysis

Univariate analysis was performed in infected and uninfected groups. Variables with *P* < 0.2 in univariate analysis were included as explanatory variables in the multivariate logistic regression analysis using a stepwise selection method. The monthly infection data were evaluated using autocorrelation analysis to assess the temporal patterns of postoperative infections. The autocorrelation function (ACF) was calculated up to a lag of 12 months to identify any seasonal or periodic patterns during infection. All data analyses were performed using the Statistical Package for the Social Sciences software SPSS version 27.0 (IBM Inc., Chicago, IL, USA).

## Results

Of 483 patients, 11 (2.3%) had an infection, including nine patients with deep infection and two with superficial infection (Table 1). Detailed microbiological profiles, including antibiotic resistance patterns, are provided in Table S1. The noninfected and infected groups consisted of 472 and 11 cases, respectively. When comparing the background factors, the albumin level was significantly lower in the infected group (4.1 ± 1.8 vs. 3.7 ± 0.8 mg/dL, *P* = 0.12), and the preoperative CRP level was significantly higher in the infected group (0.74 ± 1.8 vs. 3.2 ± 5.3 mg/dL, *p* = 0.00) (Table 2). The proportion of emergency surgeries was significantly higher in the infected group (27.8% vs. 63.6%, *P* = 0.00) (Table 3).

**Table 1:** Clinical characteristics and causative bacteria of patients with SSI.

Case	Sex	Age	Diagnosis	Surgery	Emergency Surgery	Causative bacteria	Diabetic mellitus	ASA	BMI (kg/m^2^)	CRP (mg/dL)	Albumin (mg/dL)	Surgery Time(min.)	Amount of Bleeding(ml)
1	F	70	Thoracic metastasis	PF + LM	〇	Unknown	〇	2	26.7	2.51	3.9	174	130
2	M	54	Lumbar spinal stenosis	TLIF		Unknown	〇	3	29.7	0.03	4.2	311	165
3	M	76	Cervical spine injury	PF + LP	〇	*Staphylococcus capitis*		3	21.2	1.97	3.5	145	20
4	M	78	Lumbar spinal fracture	PF		*Enterococcus faecium*	〇	3	24.9	5.43	2.3	128	50
5	F	85	Cervical spinal fracture	PF	〇	*Staphylococcus aureus*,MRSA		3	21.4	0.14	4.4	117	50
6	F	26	Lumbar disc herniation	TLIF	〇	*Staphylococcus aureus*		*1*	*20.1*	*0.42*	*4.0*	*184*	*200*
7	F	53	Lumbar spinal stenosis	TLIF		*Cutibacterium acnes*		*2﻿*	*30.6*	*0.12*	*4.3*	*250*	*265*
8	M	53	Thoracic metastasis	PF + LM	〇	*Staphylococcus aureus*		*3*	*22.4*	*16.5*	*2.6*	*255*	*355*
9	M	75	Thoracic metastasis	PF + LM	〇	*Corynebacterium striatum*	〇	*3*	*23.6*	*1.04*	*3.7*	*203*	*70*
10	M	87	Thoracic fracture	PF	〇	*Staphylococcus aureus*		*2*	*19.1*	*0.85*	*3.9*	*98*	*120*
11	M	79	Lumbar spondylolisthesis	TLIF		Unknown			26.2	0.24	4.4	22.7	520

MRSA: Methicillin-Resistant Staphylococcus aureus, PF: Posterior fusion, TLIF: Transforaminal lumbar interbody fusion, LP: Laminoplasty.

LM: Laminectomy, BMI: Body Mass Index, ASA: American Society of Anesthesiologists.

**Table 2:** Comparison of patient-related factors between the infected and noninfected groups.

	Non infection (ཎ = 472)	Infection (*n* = 11)	*P*-value
Age	69.0 ± 14.2	66.9 ± 18.4	0.98^a^
Sex (% female)	45.8	36.4	0.54^b^
Malignancy (%)	14.9	27.3	0.48^b^
BMI (kg/m^2^)	29.6 ± 13.4	24.2 ± 3.8	0.58^a^
Diabetic mellitus	18.4	33.3	0.26^b^
Skin disease(%)	1.1	0.0	0.74^b^
Dialysis (%)	2.5	0.0	0.63^b^
Smoking (%)	9.9	0.0	0.32^b^
Steroid use (%)	4.4	0.0	0.97^b^
Immunosuppressant use	5.3	22.2	0.23^b^
Albumin (mg/dL)	4.1 ± 0.6	3.7 ± 0.8	0.12^a^
Total Protein(mg/dL)	6.9 ± 0.9	6.8 ± 0.6	0.53^a^
CRP (mg/dL)	0.74 ± 0.1.8	3.2 ± 5.3	0.00^a^
ASA ≧ 3	34.8	66.7	0.31^b^
Nasal culture pathogen carriage	12.4	12.5	0.90^b^
Infection level (Cervical: thoracic: lumbar)	91:103:280	2:3:4	0.91^b^

^a^Mann–Whitney U test ^b^Chi-squared test ASA: American Society of Anesthesiologists.

BMI: Body Mass Index.

**Table 3:** Comparison of surgery-related factors between the infected and noninfected groups.

	Non infection (ཎ = 472)	Infection (*n* = 11)	*P*-value
Re-surgery (%)	8.7	22.2	0.58^b^
Emergency surgery (%)	27.8	63.6	0.00^b^
Surgery time (min)	185.9 ± 75.7	190.2 ± 66.3	0.65^a^
Number of fusion segments	3.1 ± 2.0	2.3 ± 2.1	0.22^c^
Fixation over the pelvis (%)	1.5	0.0	0.68^b^
Blood loss (mL)	260.9 ± 294.1	175.6 ± 169.9	0.28^a^
Vancomycin powder use (%)	7.4	0.0	0.72^b^

^a^Mann–Whitney U test ^b^Chi-square test c: Student t-test.

**Fig. 1:**
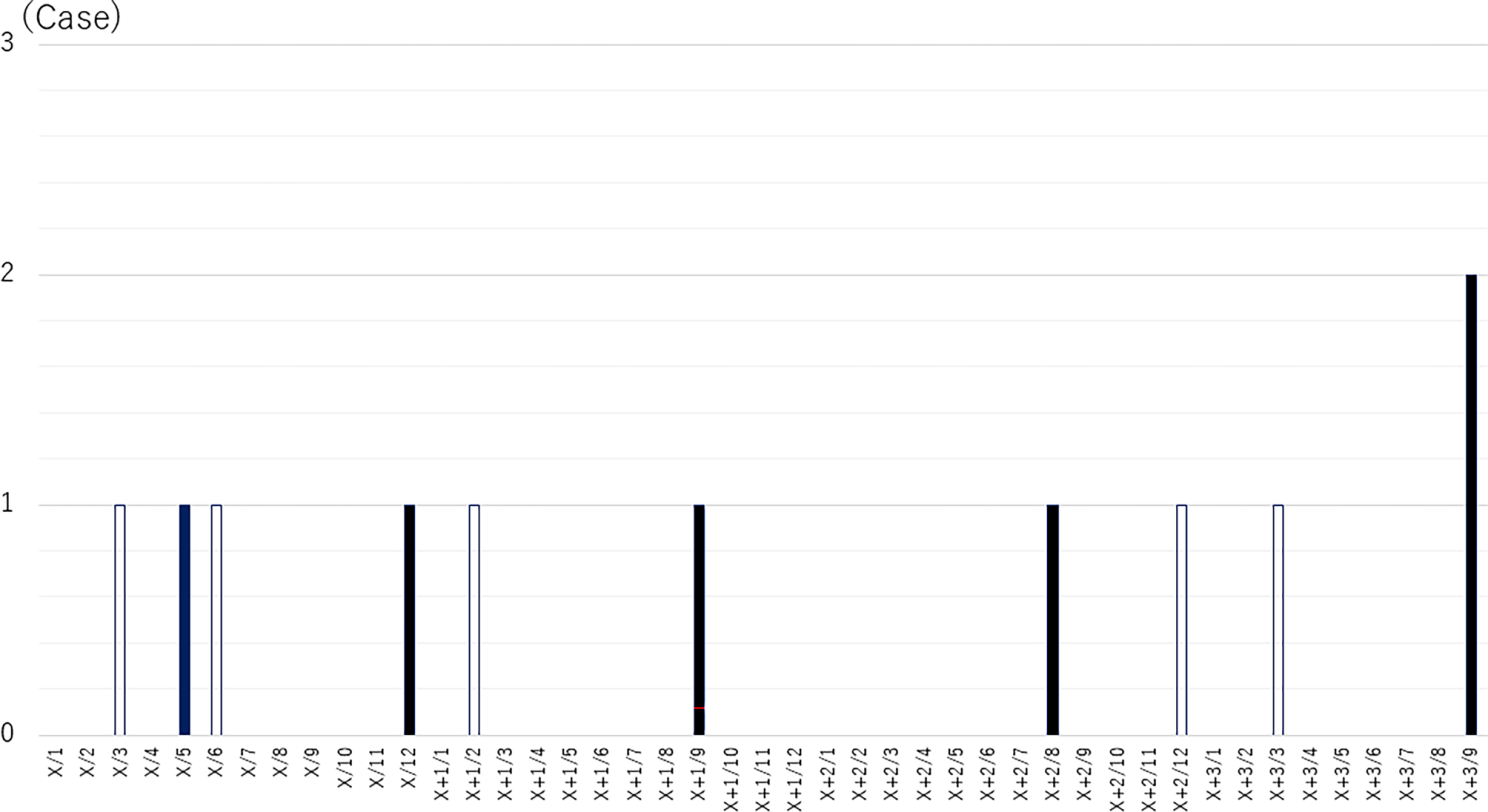
Temporal distribution of SSI cases, showing clustering within consecutive months and potential seasonal trends. (White bar: scheduled surgery, Black Ber: Emergency surgery).

**Fig. 2:**
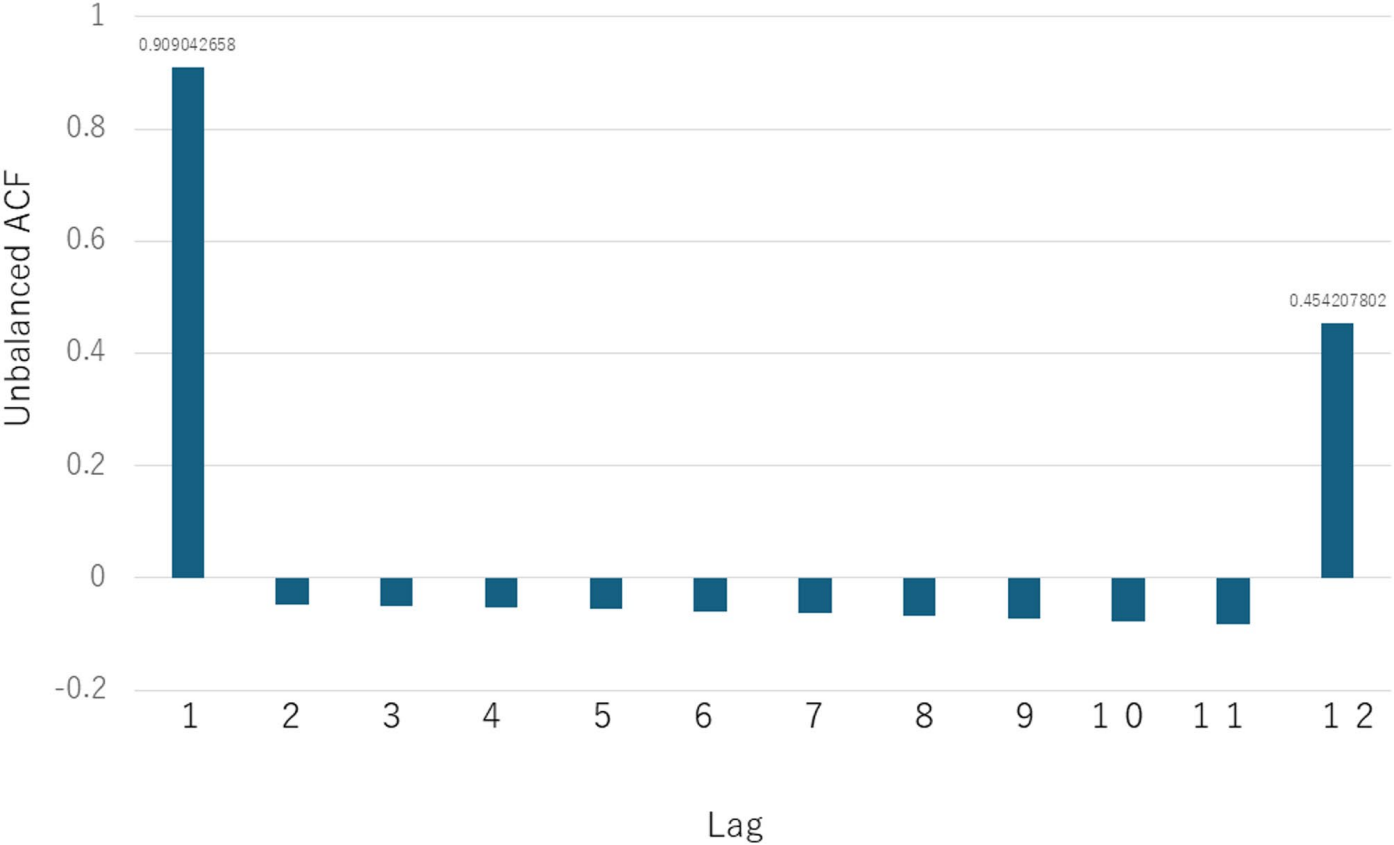
ACF analysis of the monthly SSI data revealed clustering (lag 1) and seasonal patterns (lag 12).

The variables included in the initial multivariate logistic regression model were emergency surgery, CRP level, and albumin level, showing a trend toward association in the univariate analysis (*P* < 0.2). Using stepwise selection, CRP and albumin levels were excluded during the iterative process, and only emergency surgery remained as a significant independent associated factor for SSI (odds ratio: 4.59, 95% CI: 1.322–15.942, *P* = 0.016) (Table 4). The distribution of SSI incidence across BMI categories is presented in Table 5. The occurrence of SSI did not differ significantly among the categories. Figure 1 shows the timing of infection occurrences during the study period, with infections occurring in clusters. The ACF was calculated up to a lag of 12 months to identify any seasonal or periodic patterns during infection. The results showed a high autocorrelation at lag 1 (0.909), indicating that infections tended to cluster within consecutive months. Additionally, a moderate autocorrelation at lag 12 (0.454) was observed, indicating a potential seasonal pattern, wherein infections occur at similar times annually, indicating that postoperative infections likely occur in clusters, particularly in the month following an initial infection, and may also exhibit seasonal patterns (Fig. 2).

**Table 4:** Multivariate analysis identified emergency surgery as a risk factor for SSI.

Variable	OR (Exp(B))	95% CI (Lower–Upper)	*p*-value
Step 1a (Emergency, CRP, Albumin)			
Emergency surgery	2.93	0.73–11.79	0.130
CRP positive	2.33	0.52–10.51	0.270
Albumin	0.81	0.30–2.20	0.681
Step 2a (Emergency, CRP)			
Emergency surgery	3.12	0.80–12.19	0.101
CRP positive	2.52	0.58–10.90	0.218
Step 3a (Final model)			
Emergency surgery	4.59	1.32–15.94	0.016

Variables with *P* < 0.2 in the univariate analysis were entered into the stepwise logistic regression model. CRP and albumin were excluded during the stepwise procedure, leaving emergency surgery as the only significant independent factor.

**Table 5:** Incidence of SSI by BMI category.

BMI Category	*N* (cases)	SSI (*n*)	Incidence (%)	Odds Ratio (95% CI)	*p*-value
< 18.5	34	0	0.0	–	–
18.5–24.9	300	7	2.3	–	–
25.0–29.9	120	3	2.5	1.07 (0.27–4.22)	0.92
>=30.0	29	1	3.4	1.49 (0.18–12.6)	0.71

BMI: Body mass Index.

## Discussion

We performed a cross-sectional analysis of the factors associated with SSI in spinal instrumentation surgery. The cross-sectional analysis results showed that SSI was associated with emergency surgery and indicated that once an SSI occurs, it may recur shortly, which is interesting because the possibility of postoperative infection outbreaks has not been reported.

Many risk factors for SSI have already been analyzed, including patient-related causes, such as obesity, smoking history, alcohol withdrawal, poor nutrition, diabetes mellitus, anemia, MRSA carriage, history of infection, skin disease, renal function, and poor respiratory function^3,4,15^. Obesity has been identified as a risk factor; however, obesity was defined as a BMI of ≥ 30.

in previous studies^16,17^. In our cohort, BMI did not emerge as a significant associated factor for SSI. As shown in Table 5, the incidence of SSI was uniformly low across BMI categories, with no cases observed among underweight patients and only one case among those with BMI ≥ 30. This limited number of patients at the extremes of BMI likely reduced the statistical power to detect an association. Therefore, while obesity has been reported as a risk factor in other surgical fields, our findings suggest that its impact could not be adequately assessed in this study due to the restricted distribution of BMI values. Surgical factors for spinal instrumentation include prolonged surgery^6,9^ and using osteotomy^3^, long fusion^5,6,18^, fixation over the sacrum or pelvis^7,8^. In our case, we had little intervertebral fusion and almost no fusion involving the pelvis, so these factors may not have been extracted as being related to infection. Dubory et al. examined the risk factors for infection in surgery for relatively urgent spinal injuries and found that age, diabetes, and surgery lasting > 3 h were risk factors, although any causes specific to emergency surgery were not mentioned^19^. By contrast, these factors have been clarified, and various infection control measures have been proposed. Thus, surgeons have considered several strategies to prevent SSIs. First, the patient’s condition should be controlled, such as blood sugar control and checking nasal cultures for MRSA^20^.

Second, perioperative strategy, such as irrigation saline with povidone-iodine, should follow the guidelines of the Centers for Disease Control and Prevention^10^, regularly antimicrobial administration and using suture method antimicrobial materials.

In our study, emergency surgery was an independent associated factor. In spinal instrumentation surgery, infections can generally lead to significant complications or even life-threatening issues. Therefore, various countermeasures have been developed. Yamada et al. reported that the postoperative spinal infection rate was reduced from 3.8% to 0.7% by establishing certain infection control measures in spinal instrumentation surgery^21^. However, some measures cannot be implemented in emergency surgeries. Additionally, patients undergoing emergency surgeries are often in poor conditions due to factors, such as trauma or metastatic tumors causing paralysis. Although no studies have reported the relationship between emergency surgery and infection in spinal surgery, in orthopaedic trauma surgery, a higher incidence of SSI has been reported^2^. Not only local tissue vulnerability but also the fact that procedures are often performed before sufficient infection surveillance and preparation can be secured may contribute to this increased risk. Furthermore, obesity and diabetes have also been identified as significant risk factors for SSI in trauma surgery^3^, conditions that are difficult to optimize preoperatively because of the urgent nature of these procedures. A similar situation is frequently encountered in emergency spinal surgery, where urgent intervention precludes adequate preoperative preparation^22^. These factors may partly explain why emergency surgery has been identified as an independent risk factor for SSI (OR 4.59, *P* = 0.016), and why both trauma surgery and emergency spine surgery carry inherently higher risks of SSI compared with elective procedures.

Statistical analysis also provided important insights. The autocorrelation function (ACF) showed significant correlations at lag-1 and lag-12. Lag-1 suggests that once an infection occurred, subsequent infections tended to cluster in time, indicating possible lapses in infection control. Lag-12 suggests a seasonal trend, potentially linked to fluctuations in patient volume or resource allocation. These findings, though exploratory, emphasize the need for continuous surveillance and timely reinforcement of prevention protocols.

Previous studies, such as the multi-institutional study by Durkin et al., have reported seasonal variations in SSI occurrences, with higher rates in summer. This report stated that 1.29% of the 33,093 instrumentation surgeries resulted in infection, although *Staphylococcus aureus* caused more infections in the summer^23^. Gruskay et al. also reported many cases of infection in the summer, but in addition to seasonal factors, the quality of infection control measures may have decreased due to personnel changes, etc., and the so-called “July Effect” was considered^24^. We found that once SSI occurs, subsequent infections occur shortly, regardless of the season. Although we were unable to elucidate the exact reason for repeated infections, the clustering of infections suggests insufficient infection control measures, particularly during consecutive emergency surgeries. Some studies based on the perspective of nurses reported that heavy workload lowers the quality of medical care and increases the incidence of hospital-acquired infections^25^. The quality of organizational countermeasures can degrade under persistent pressure, raising the possibility of slight lapses in infection prevention measures due to human error during emergencies.

Our study has several limitations. First, the sample size was small compared with other reports. Many risk factors for postoperative spinal infections have already been analyzed through large-scale studies and meta-analyses. However, because this was a single-institution study, it uniquely highlighted the phenomenon of recurrent infections within a short period, which may not be detectable in multi-institutional or database-driven studies. Second, the study might have had selection bias. As a single-institution study, the characteristics of the surgeries performed may have influenced the results. For example, surgeries involving extensive spinal fixation or osteotomy, such as adult spinal deformity procedures, were less represented in this study, which might have skewed the analysis of infection risk factors. Despite comprehensive analyses and countermeasures, we should recognize and address occasional lapses in infection prevention measures. This study indicates that infection control practices should be reviewed, particularly under emergency conditions. Multi-institutional studies are required to verify whether similar phenomena occur elsewhere.

This study examined the associated factors for infections in spinal instrumentation surgeries. Emergency surgery was identified as a significant risk factor. Additionally, subsequent infections were likely to recur once an infection occurred, indicating possible lapses in infection control measures. These findings underscore the importance of regularly reviewing and improving infection prevention protocols. In particular, when emergency surgeries are repeatedly required, surgeons should recognize that surveillance opportunities are inherently limited, which may predispose to infection clustering. Under these conditions, reconfirming that infection prevention protocols are applied consistently and effectively may be more practical than relying on routine surveillance alone.

## Supplementary Information

Below is the link to the electronic supplementary material.

Supplementary Material 1

## Data Availability

The data that supports the findings of this study are available from the corresponding author, Shinji Tanishima, upon reasonable request.
