# Supplementary material for: Associated factors for surgical site infection with spinal instrumentation surgery
Source: Sci Rep. 2025 Nov 18;15:40543. doi: 10.1038/s41598-025-24209-y (PMC12627094; doi:10.1038/s41598-025-24209-y)
Supplement: Supplementary file 1 — Supplementary Material 1 [file 41598_2025_24209_MOESM1_ESM.docx]

Title: Associated factors for surgical site infection with spinal instrumentation surgery

Shinji Tanishima*, Tokumitsu Mihara, Chikako Takeda, Satoshi Fujiwara, Hideki Nagashima

Division of Orthopedic Surgery, Department of Sensory and Motor Organs, School of Medicine, Faculty of Medicine, Tottori University, Tottori, Japan

Corresponding Author

Shinji Tanishima

Division of Orthopedic Surgery, Department of Sensory and Motor Organs, School of Medicine, Faculty of Medicine, Tottori University

36-1 Nishi-cho, Yonago, Tottori 683-8504, Japan

Tel: +81-859-38-6587

Fax: +81-859-38-6589

E-mail: shinjit@tottori-u.ac.jp

ORCID: <https://orcid.org/0000-0002-8137-4658>

Supplementary TableS1: Microbiological profiles and antibiotic resistance patterns of patients with SSI

| Case No. | Pathogen identified | Resistance profile |
| --- | --- | --- |
| 1 | Staphylococcus capitis | MRS |
| 2 | Enterococcus faecium | – |
| 3 | Staphylococcus aureus | MRSA |
| 4 | Staphylococcus aureus | – |
| 5 | Cutibacterium acnes | – |
| 6 | Staphylococcus aureus | – |
| 7 | Corynebacterium striatum | – |
| 8 | Staphylococcus aureus | – |
| 9 | Unknown | – |
| 10 | Unknown | – |
| 11 | Unknown | – |

MRS : methicillin-resistant staphylococci (including coagulase-negative staphylococci).

MRA :methicillin-resistant Staphylococcus aureus.

“–”: no resistance reported or not applicable.
